# Study on nanometric cutting of germanium by molecular dynamics simulation

**DOI:** 10.1186/1556-276X-8-13

**Published:** 2013-01-05

**Authors:** Min Lai, Xiaodong Zhang, Fengzhou Fang, Yufang Wang, Min Feng, Wanhui Tian

**Affiliations:** 1 State Key Laboratory of Precision Measuring Technology & Instruments, Centre of MicroNano Manufacturing Technology, Tianjin University, Tianjin, 300072, China; 2 School of Physics, Nankai University, Tianjin, 300071, China

**Keywords:** Molecular dynamics simulation, Germanium, Extrusion, Phase transformation, Amorphization

## Abstract

Three-dimensional molecular dynamics simulations are conducted to study the nanometric cutting of germanium. The phenomena of extrusion, ploughing, and stagnation region are observed from the material flow. The uncut thickness which is defined as the depth from bottom of the tool to the stagnation region is in proportion to the undeformed chip thickness on the scale of our simulation and is almost independent of the machined crystal plane. The cutting resistance on (111) face is greater than that on (010) face due to anisotropy of germanium. During nanometric cutting, both phase transformation from diamond cubic structure to β-Sn phase and direct amorphization of germanium occur. The machined surface presents amorphous structure.

## Background

Monocrystalline germanium is widely used in the fields of semiconductors, infrared optics, high-frequency electronics, and so on. Single-point diamond turning is usually adopted to achieve high surface finish and intricate features. However, it is hard to obtain perfect optical quality and complex surfaces for monocrystalline germanium because of its brittle nature. Therefore, understanding the mechanism of nanometric cutting and machined surface characteristics is of great significance in manufacturing high quality germanium components.

Since 1990s, Shimada et al. have conducted a series of investigations on the mechanism of nanometric cutting of single crystals by molecular dynamics (MD) simulation. They found dislocations generated during nanometric cutting of aluminum and copper [1,2]. The single crystal silicon was removed in ductile mode when the depth of cut decreased to nanoscale, and amorphous silicon on machined surface was observed after nanometric cutting [3,4]. Komanduri et al. studied the effect of crystal orientation on the nature of cutting deformation for copper and aluminum by molecular dynamics simulation [5-7]. They concluded that the phase transformation from a diamond cubic to β-Sn structure appeared in the case of nanometric cutting on silicon. Fang et al. proposed the extrusion model for cutting materials at nanometric scale, indicating that the conventional cutting theory could no longer explain the mechanism of nanoscale cutting [8-11]. The process of nanocutting was affected by the tool-edge radius, and monocrystalline crystal silicon transformed into polycrystal and amorphous structure during and after nanocutting.

Previous investigations indicate that the deformation mechanism of single crystal copper and aluminum during nanometric cutting is mainly the formation and extension of dislocations. However, silicon is removed in ductile mode; phase transformation and amorphization are the main deformations during nanometric cutting, observed by molecular dynamics simulation. At present, study on the nanometric cutting of germanium by molecular dynamics simulation has rarely been reported. In this paper, large-scale three-dimensional MD simulations are conducted to study the nanometric cutting of germanium. Attentions are focused on the material flow, cutting force and energy, crystal orientation effect, and surface-subsurface deformation.

## Methods

### MD simulation method

**Figure 1:**
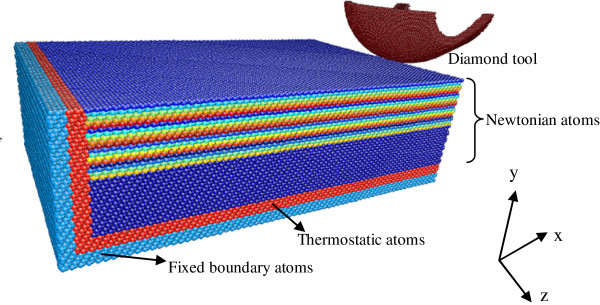
Model of molecular dynamics simulation.

Figure 1 shows the three-dimensional MD simulation model of nanometric cutting. The work material is a monocrystalline germanium with a size of 45 × 27 × 12 nm. The workpiece consists of three kinds of atoms: boundary atoms, thermostat atoms, and Newtonian atoms. The several layers of atoms on the bottom and exit end of the workpiece keep the position fixed in order to prevent the germanium from translating, which results from the cutting force. It is a widely acceptable boundary condition for MD simulation model of nanometric cutting and scratching [12,13]. The several layers of atoms neighboring the boundary atoms are kept at a constant temperature of 293 K to imitate the heat dissipation in real cutting condition, avoiding the bad effects of high temperature on the cutting process. The rest atoms belong to the Newtonian region, which is the machined area. Their motion obeys the classical Newton’s second law, and they are the object for investigating the mechanism of nanometric cutting.

**Figure 2:**
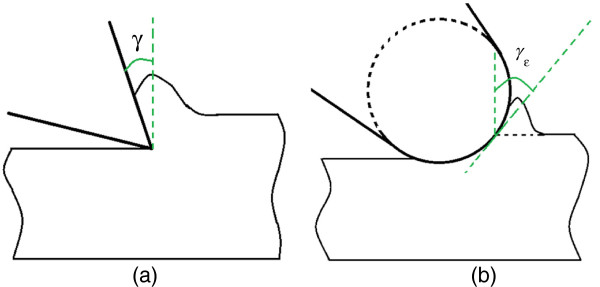
**Different rake angles.** (**a**) Positive rake angle (*γ*) and (**b**) effective negative rake angle (*γ*_e_) in nanometric cutting.

Since the depth of cut is usually smaller than the tool-edge radius in real nanometric cutting, the effective rake angle is always negative regardless of whether nominal rake angle is negative or not [10]. Positive rake is, by definition, the angle between the leading edge of a cutting tool and a perpendicular to the surface being cut when the tool is behind the cutting edge. Otherwise, the rake angle is negative, as shown in Figure 2.

In this paper, the tool is modeled as the shape of a real cutter, which was firstly conducted by Zhang et al. [14], as shown in the Figure 1. The tool-edge radius is 10 nm, and the undeformed chip thickness is set as 1 to 3 nm in order to get large negative rake angle, which agrees with the condition of the real nanocutting.

For covalent systems, the Tersoff potential [15,16] was used to depict the interaction among the germanium atoms of the substrate, similar with the silicon [7,12-14]. Usually, the interaction between rigid diamond tool and silicon atoms is described by the Morse potential as follows:

(1) $$E\left(r\right)=\mathrm{De}\left[e^{-\mathrm{q\alpha}\left(r-r_{0}\right)}-qe^{-\alpha \left(r-r_{0}\right)}\right]$$

**Figure 3:**
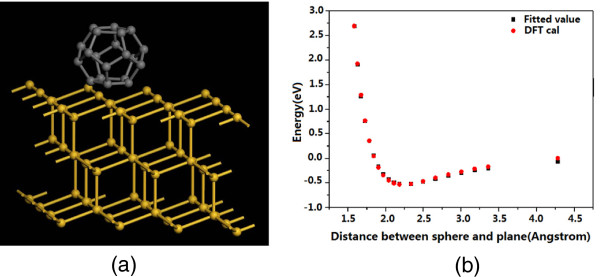
**Potential between germanium atoms and diamond atoms.** (**a**) Schematic diagram of simulation model for germanium plane and carbon sphere interaction; (**b**) simulated and fitted energy values when the distance between sphere and plane changes.

The E(r) is the pair potential energy, r0 and r are the equilibrium and instantaneous distances between two atoms, respectively, De and α are the constants determined on the basis of the physical properties of the materials, q is a constant equal to 2. Since the crystal structure and nature of monocrystalline germanium are similar with that of monocrystalline silicon, the Morse potential is selected to depict the interaction of tool atoms and germanium atoms. However, no literatures have offered the parameters of Morse potential between germanium atoms and carbon atoms. In this study, computer simulation is used to obtain the relevant parameters, as shown in Figure 3a. The cluster of carbon atoms is treated as the atoms of diamond tool, and the several layers of monocrystalline germanium are deemed to be the substrate. The interaction energy is calculated by the first principle calculation when the distance between the sphere and plane changes. Figure 3b shows the calculated and fitted values of interaction energy. The parameters of the Morse potential can be achieved from the fitted energy curve. Details about workpiece and simulation are listed in Table 1.

**Table 1:** Model condition and simulation parameters

**Condition**	**Parameter**
Work material	Germanium
Lattice constant	*a* = 5.657 Å
Potential for germanium	Tersoff potential
Potential of C-Ge	Morse potential
	*De* = 0.125778 eV, *α* = 2.58219 Å^−1^, 0 *r*_0_ = 2.2324 Å
Work dimensions	45 × 27 × 12 nm
Tool-edge radius	10 nm
Tool-nose radius	10 nm
Tool clearance angle	15°
Cutting direction	$\left[\overset{-}{1}00\right]$ on (010) surface
	$\left[\overset{-}{2}11\right]$ on (111) surface
Depth of cut	1, 2, 3 nm
Cutting speed	400 m/s
Bulk temperature	293 K

## Results and discussion

### Model of nanometric cutting

**Figure 4:**
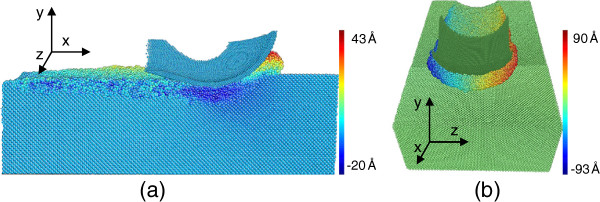
**Material flow in nanometric cutting.** (**a**) Cross-sectional view of the atom’s displacement in *y* direction; (**b**) atom’s displacement in *z* direction.

Figure 4 shows the material flow of germanium in nanometric cutting. The atoms in Figure 4a are colored by their displacement in *y* direction. It can be seen that a part of the machined workpiece atoms flows up to form a chip, and others flow downward along the tool face to form the machined surface, resulting in the negative displacement in *y* direction of finished surface atoms. The boundary of material flow is named as stagnation region [10,17]. The germanium atoms pile up by extruding in front of the tool and side-flowing along the tool face, which are called extrusion and ploughing, as shown in Figure 4b. The material flow of the monocrystalline germanium during nanometric cutting is the same as that of copper and silicon [10,17].

**Figure 5:**
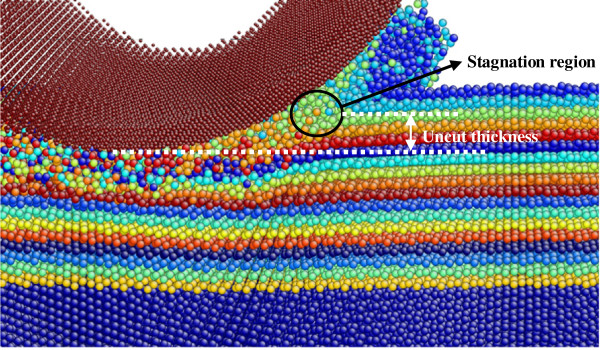
**Cross-sectional view of nanometric cutting along [**$\overset{―}{2}11$**] on (111) crystal plane.**

Figure 5 shows the cross-sectional view of the stable phase of nanometric cutting along the feeding direction when machining along $\left[\bar{2}11\right]$ on (111) surface. The surface and subsurface of germanium are colored by different layers in order to monitor the motion of every atomic lay, so as to observe the location of stagnation region. The undeformed chip thickness is 2 nm. It can be seen that the demarcation of material flow locates on the rake face instead on the tool bottom. The atoms in this region neither flow up to accumulate as a chip nor flow downward to form the machined surface, which seem ‘stagnated’. The depth from the bottom of the tool to the stagnation region is defined as ‘uncut thickness’ [17].

**Figure 6:**
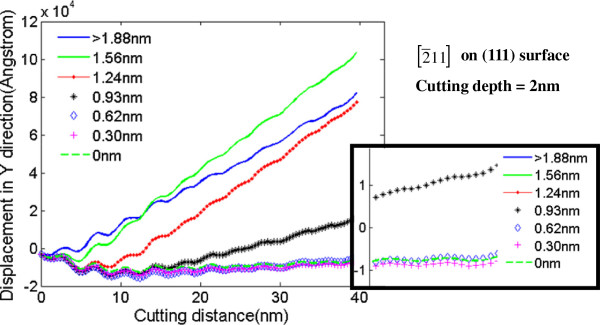
**Displacement vector sum of each layer in *y *direction.**

Figure 6 shows the displacement vector sum curve of every layer in the surface and subsurface of workpiece during nanometric cutting. The position of stagnation region can be acquired from the value of displacement vector sum, which means that the range from minimum positive value to maximum negative value is considered as the location of stagnation region. Because of the radius of neighboring crystal layers, the uncut thickness should be a range rather than a certain value, as displayed in Table 2.

**Table 2:** The uncut thickness in different combinations of depth of cut and lattice plane

**Cutting direction**	**Cutting depth (nm)**	**Uncut thickness (nm)**
$\left[\overset{―}{1}00\right]$ on (010) surface	1	0.45-0.58
	2	0.87-1.01
	3	1.23-1.38
$\left[\overset{―}{2}11\right]$ on (111) surface	1	0.35-0.58
	2	0.68-0.93
	3	1.07-1.28

**Figure 7:**
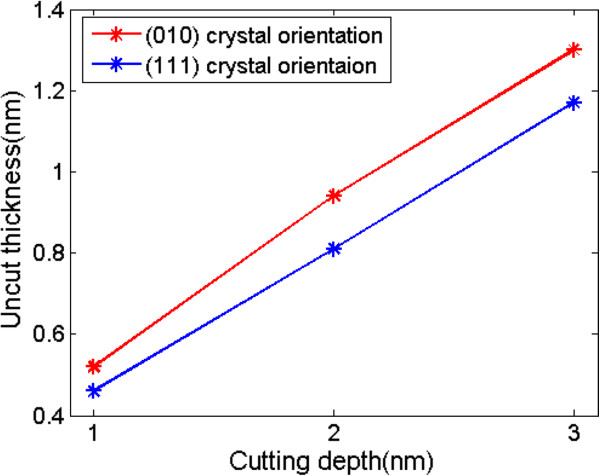
**The uncut thickness.** In different depths of cut when machined surfaces are (010) and (111) plane, respectively.

Figure 7 shows the average uncut thickness in different undeformed chip thicknesses when machined surfaces are (010) and (111) plane, respectively. The uncut thickness increases with an increase in undeformed chip thickness. With the same combination of cutting direction and crystal orientation, the uncut thickness is nearly proportional to the undeformed chip thickness on our simulation scale [17]. The uncut thickness of machining on (010) crystal orientation is about 0.1 nm bigger than that on (111) crystal orientation with the same undeformed chip thickness, which means that the difference can be ignored considering the interplanar distance.

### Cutting force and energy

**Figure 8:**
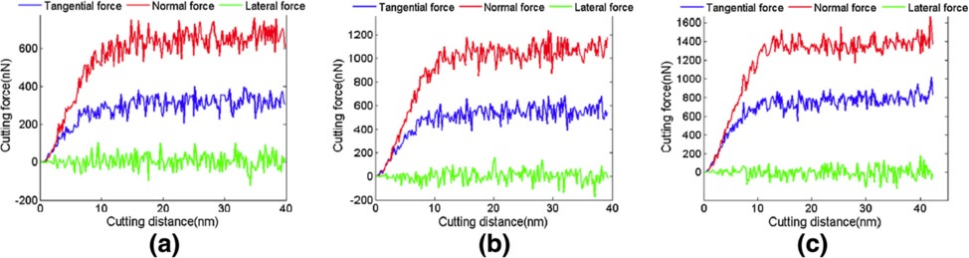
**Cutting forces.** Undeformed chip thickness is (**a**) 1, (**b**) 2, and (**c**) 3 nm.

The cutting force derives from the interaction between the tool and material atoms in the molecular dynamics simulation of nanometric cutting. Since it has a great influence on the surface finish, tool wear, etc., the cutting force is monitored during the machining process. The sum of force vector on three axes directions, namely *Fx*, *Fy*, and *Fz*, are defined as tangential force, normal force, and lateral force, respectively. When machining along $\left[\bar{1}00\right]$ on (010) surface with cutting depth of 1 nm, 2 nm and 3 nm, the calculated cutting forces including tangential, normal, and lateral forces, are indicated in Figure 8. On the initial stage of the cutting process, the tangential and normal forces start to increase rapidly until the distance of cutting increases to about 10 nm. From then on, the increasing rate of the cutting force starts to slow down until reaching the steady stage of the cutting process, on which the cutting forces always undulate around the equilibrium value. The lateral force fluctuates around zero because the two side forces of the tool counteract with each other. The fluctuation in cutting force derives from the thermal motion of atoms and the undulation of energy, which results from the deformation of crystal structure during nanometric cutting.

The average tangential and normal forces during the steady stage are calculated when cutting directions are $\left[\bar{1}00\right]$ on (010) surface and $\left[\bar{2}11\right]$ on (111) surface, respectively. Due to the numbers of contacting atoms are different with the various combinations of cutting depth and machining direction, the tangential and normal forces cannot be used to estimate the cutting resistance directly. Usually, the frictional coefficient is a criterion to estimate the machining resistance, which is defined as the ratio of average tangential force to normal force during the steady stage. All the average cutting forces and frictional coefficients are listed in Table 3.

**Table 3:** Average cutting force and frictional coefficient with different undeformed chip thickness

**Cutting direction**	**Cutting depth (nm)**	**Tangential force (nN)**	**Normal force (nN)**	**Frictional coefficient**
$\left[\overset{―}{1}00\right]$ on (010) surface	1	315.3	647.5	0.487
$\left[\overset{―}{2}11\right]$ on (111) surface	1	342.5	659.1	0.520
$\left[\overset{―}{1}00\right]$ on (010) surface	2	550.7	1056.9	0.521
$\left[\overset{―}{2}11\right]$ on (111) surface	2	592.4	1058.5	0.560
$\left[\overset{―}{1}00\right]$ on (010) surface	3	778.0	1360.4	0.572
$\left[\overset{―}{2}11\right]$ on (111) surface	3	850.4	1372.8	0.619

**Figure 9:**
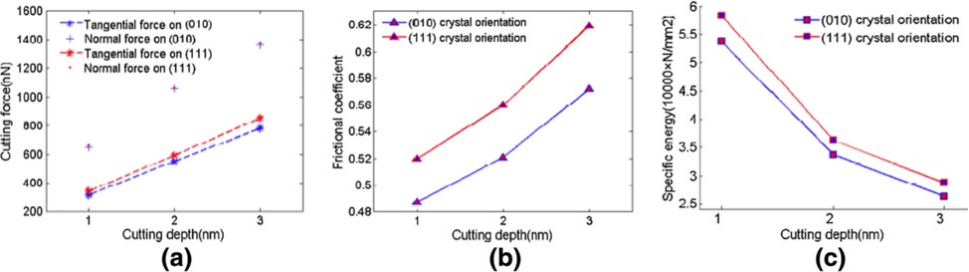
**Cutting characteristics variations.** (**a**) Cutting force, (**b**) frictional coefficient, and (**c**) specific energy. The crystal orientations are on (010) plane and (111) plane.

In the same crystal orientation, the tangential and normal forces increase with an increase in undeformed chip thickness as expected. Meanwhile, the frictional coefficient also augments, which means the cutting resistance increases. With the same undeformed chip thickness, the tangential force on (111) crystal face is greater than that on (010) crystal face, and the difference becomes bigger when the undeformed chip thickness increases. However, the average normal forces for both of them are almost the same with the same undeformed chip thickness. It implies that the cutting resistance of nanometric cutting along $\left[\bar{2}11\right]$ on (111) surface is greater than that along $\left[\bar{1}00\right]$ on (010) surface, as shown in Figure 9a,b. Except for the heat dissipation, the energy dissipations for nanometric cutting are mainly the amorphization of chip and machined surface when undeformed chip thickness is 3 nm. (111) plane of germanium has a bigger atomic planar density than (100) plane, so the cutting force of machining on (111) plane is greater than that on (100) plane.

Figure 9c shows the variation in specific energy with the change of depth of cut. The specific energy decreases with an increase in undeformed chip thickness, which can be explained by the size effect [7]. This phenomenon depends on several factors such as material strengthening, extrusion and ploughing due to finite edge radius, material separation effects, and so on.

### Surface and subsurface deformation

Germanium and silicon belong to the group IV elements, of which the single crystals are important technological materials with a wide range of applications in semiconductor field, and their natures are similar in many aspects. With an increase in pressure, both experimental and theoretical investigations show that phase transformation in germanium from its diamond cubic structure to the metallic β-Sn structure would take place under pure hydrostatic pressure of about 10 GPa [18]. On slow pressure release, a simple tetragonal phase with 12 atoms per unit cell (ST12) [19,20] forms, while a metastable body-centered cubic structure with eight atoms per unit cell (denoted BC8) [21] forms on fast pressure release. Previous investigations show that the phase transformation from diamond cubic phase to the β-Sn phase of silicon occurs during nanometric cutting, and the amorphous silicon is observed after machining.

**Figure 10:**
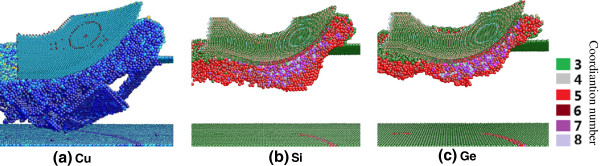
**Cross-sectional view of subsurface deformation of copper, silicon, and germanium during nanometric cutting.** The perfect FCC structure and diamond cubic structure are hidden.

Figure 10 displays the snapshots of nanometric cutting on cooper, silicon, and germanium, respectively. The atoms in Figure 10a are colored according to the value of the centro-symmetric parameter, and the atoms with centro-symmetric parameter less than 3 are hidden, representing the perfect FCC structure including elastic deformation [22,23]. It can be seen that the dislocations extending into the material are the dominant deformations for copper during nanometric cutting. Most of the dislocations are initially parallel to {111} planes [17]. The atoms in Figure 10b,c are colored according to their coordination number, and the fourfold coordinated atoms far away from the machined region are hidden, which indicate the diamond cubic phase and its distorted structure. The coordination number and atomic bond length are usually used to identify the structural phase formation during nanoindentation and nanometric cutting of silicon [24-26]. Generally, in the case of silicon and germanium, the atoms with coordination number of 4 indicate a covalent bonded system with a diamond cubic structure. The sixfold coordinated atoms are thought as the β-Sn phase, and the fivefold coordinated atoms indicate the bct5 structure, which is considered as an intermediate in the formation of sixfold-coordinated β-Sn phase [16,27]. The atoms with coordination number of 7 or more may indicate the complete amorphous structure under pressure, and the threefold or twofold coordinated atoms are indicative of the dangling bonds on the surface and sides of the work material [7,16]. It can be seen from Figure 10b that the phase transformation and amorphization instead of dislocation formation are the dominant deformations on machined surface and subsurface. The mechanism of nanometric cutting of germanium is similar with that of silicon from the snapshot shown in the Figure 10c.

**Figure 11:**
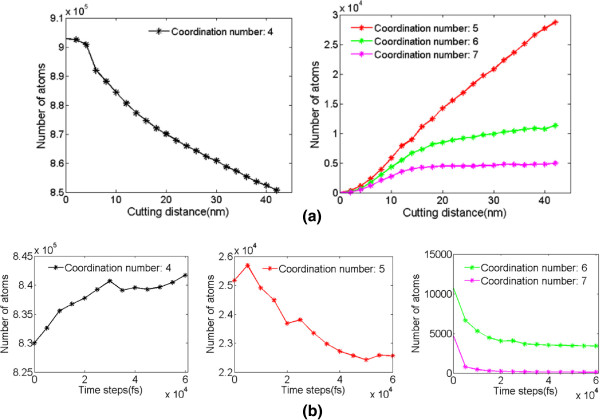
**The atomic coordination numbers.** (**a**) During cutting process and (**b**) relaxing after the cutting process.

**Figure 12:**
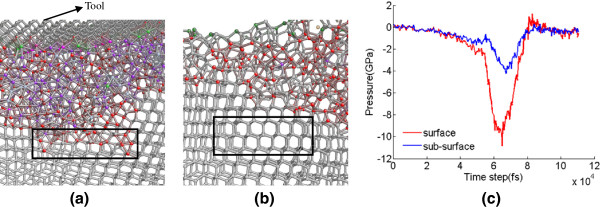
**Surface and subsurface structures of germanium.** (**a**) During cutting and (**b**) after cutting, while atoms are colored according to the coordination number; (**c**) pressure in machined surface and subsurface.

The change of coordination number for germanium atoms during nanocutting is recorded, as displayed in Figure 11. During the nanometric cutting, the numbers of fivefold and sixfold coordinated atoms increase while the number of fourfold coordinated atoms decreases, which means that the phase transformation from diamond cubic structure to β-Sn phase occurs. After cutting and then relaxing for a while, the numbers of sixfold and sevenfold coordinated atoms decrease rapidly until they settle out, and the sevenfold coordinated atoms seem to disappear. Meanwhile, the number of fivefold coordinated atoms increases slightly on initial stage and then decreases rapidly. The reason is that the fivefold coordinated atoms are the transitory stage for sevenfold and sixfold coordinated atoms transforming back to fourfold coordinated atoms. As a result, the number of fourfold coordinated atoms increases after cutting. Description above indicates that the atoms in deformed layer of machined surface have a mix of four and five neighbors and few six neighbors, which is proved to be the feature of amorphous germanium in the molecular dynamic simulation [28,29]. The same result can be obtained from Figure 12b, in which the machined surface presents amorphous structure, similar with silicon as stated by Cheong and Zhang [30].

Figure 12a,b show the crystal structure of surface and subsurface for germanium during and after nanocutting, respectively. When the tool cuts on the surface to get the maximum stress, the distorted diamond cubic structure and other structures with fivefold or sixfold coordinated atoms are observed in the subsurface region shown in black rectangle, and they all transform back to the diamond cubic structure with coordination number of 4 after stress release. In the case of deformed region above it, the high-pressure disordered structures form amorphous germanium instead of recovering back to the diamond cubic structure after nanometric cutting. Whether the phase transformation or amorphization would take place depends on the pressure. For example, the threshold pressure inducing the phase transformation from diamond cubic structure to Ge-II and to ST12-Ge on pressure release is about 12 GPa [31]. Therefore, the pressure of the two regions shown in the Figure 12a,b during the cutting process is calculated, as displayed in Figure 12c. The maximum pressure in subsurface region (black rectangle) is about 4 GPa, which is less than the threshold pressure of phase transformation from diamond cubic structure to β-Sn phase. However, the maximum pressure produced during machining in machined surface region (above the black rectangle) is about 11 GPa, more than the critical pressure of phase transformation from diamond cubic structure to β-Sn phase, but less than 12GPa, which means that the phase transformation from β-Sn structure to ST12-Ge on pressure release would not happen. As a result, the amorphization of germanium occurs after pressure release.

**Figure 13:**
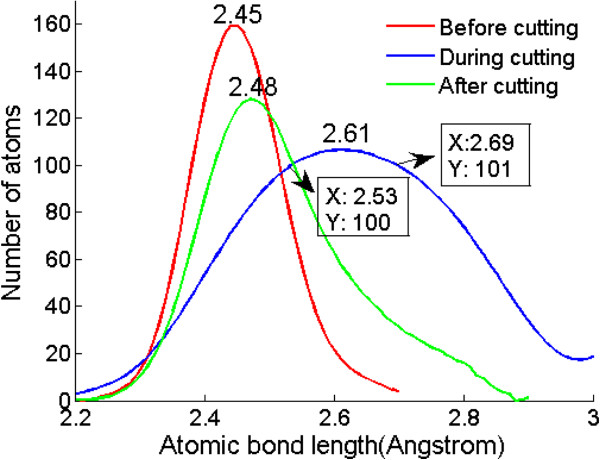
Atomic bond length distribution.

For further investigation of surface and subsurface deformation, the atomic bond length distribution before, during, and after machining are calculated, respectively, as shown in Figure 13. Before cutting, the peak value of atomic bond length is about 2.45 Å, close to the bond length of germanium diamond cubic structure of 2.445 Å [32]. When the tool is cutting on the surface, the stress of the region beneath the cutter in the material is the greatest, inducing the phase transformation from diamond cubic structure to β-Sn phase. The β-Sn structure of germanium has two bond lengths of 2.533 and 2.692 Å [32]. It can be seen from the blue line that the peak value of atomic bond length increases to 2.61 Å and a significant increase in the number of atoms with interatomic distance of 2.53 to 2.69 Å occurs, which proves the phase transformation mentioned above. The broaden bond length distribution also indicates other complicated amorphization under high pressure, such as the structure with sevenfold or higher coordinated atoms. After machining, the stress releases to a certain degree, the distribution of atomic bond length becomes centralized again, and the peak locates at about 2.48 Å. Amorphous germanium has short-range ordered and long-range disordered structures, and its nearest-neighbor distance is around 2.48 to 2.49 Å in molecular dynamic simulations when applying Stillinger-Weber and Tersoff potential [28,29]. Thus, the snapshots of machined surface structure and the peak value of atomic bond length indicate that the deformed layers of machined surface are amorphous germanium.

## Conclusions

Three-dimensional MD simulations are conducted to study the nanometric cutting of germanium. The material flow, cutting force, and specific energy with different machined faces and depths of cut are studied. The deformations of surface and subsurface during and after cutting process are discussed. The conclusions can be drawn as follows:

(1) The material flow of nanometric cutting on monocrystalline germanium is the same with that on cooper and silicon, which has extrusion and ploughing. The stagnation region is also observed.

(2) On the same crystal plane, the uncut thickness is in proportion to the depth of cut on the scale of our simulation. However, with the same undeformed chip thickness, the uncut thickness is almost the same on different machining crystal plane.

(3) The cutting force and frictional coefficient increase with an increase in the undeformed chip thickness, while the specific energy decreases because of the size effect. With the same undeformed chip thickness, the cutting resistance of machining on (111) surface is greater than that on (010) surface.

(4) Monocrystalline germanium undergoes phase transformation from diamond cubic structure to β-Sn phase, and direct amorphization with the pressure derives from the cutting of tool. The surface presents amorphous structure after machining, while some parts of subsurface recover back to distorted diamond cubic structure.

## Competing interests

The authors declare that they have no competing interests.

## Authors’ contributions

FF conceived the research work, coordinated the collaboration, and participated in the analyses. ML carried out the molecular dynamics simulations of nanometric cutting of germanium and analyzed the simulation results. XZ participated in its design, coordination, and analyses. YW, MF, and WT carried out the simulations of getting the parameters of the Morse potential. All authors read and approved the final manuscript.

## Authors’ information

ML is a Ph.D. student in the Centre of Micro/nano Manufacturing Technology (MNMT) at Tianjin University, studying in the mechanism of ultra-precision machining. XZ is an associate professor in MNMT at Tianjin University. His research interests include ultra-precision machining and metrology, freeform optics manufacture and applications. FF is a professor in MNMT, working in the areas of optical freeform manufacturing, micro/nano machining, ultra-precision machining and metrology. He is the editor-in-chief of the International Journal of Nanomanufacturing, the president of the International Society for Nanomanufacturing, and a fellow of the International Academy for Production Engineering. YW is a professor of Physics at Nankai University. Current research interests include surfaced enhanced Raman spectra, light scattering of nanoparticles and first principles calculation of materials. MF is working at Nankai University as a technician with the research objective in investigating the electronic, magnetic, and thermodynamic properties of materials using first-principles calculation, potential model, and Monte Carlo simulation. WT is studying as a masters student in optics at Nankai University.
